# Transformation of Peripheral Sexual Precocity to Central Sexual Precocity Following Treatment of Granulosa Cell Tumor of the Ovary

**DOI:** 10.7759/cureus.22676

**Published:** 2022-02-28

**Authors:** Prashant M Gaikwad, Soumik Goswami, Nilanjan Sengupta, Arjun Baidya, Niladri Das

**Affiliations:** 1 Endocrinology and Diabetes, Nil Ratan Sircar Medical College, Kolkata, IND; 2 Endocrinology, Nil Ratan Sircar Medical College, Kolkata, IND

**Keywords:** gnrh-dependent precocious puberty, triptorelin stimulation test, secondary central precocious puberty, peripheral cause of precocity, granulosa cell tumor, central precocious puberty

## Abstract

Juvenile granulosa cell tumor leading to isosexual peripheral precocious puberty is a well-known association. Here, we report a rare case of central precocious puberty secondary to granulosa cell tumor of the ovary. A five-year and five-month-old girl presented with a history of progressive enlargement of bilateral breasts and intermittent vaginal spotting, associated with growth acceleration. Elevated estradiol and suppressed serum follicle-stimulating hormone were found on investigation. Additionally, abdominal and pelvic ultrasonography was suggestive of a right ovarian mass, which proved to be a juvenile granulosa cell tumor on histopathology and immunohistochemistry, leading to a diagnosis of peripheral precocious puberty secondary to granulosa cell tumor of the ovary. One and a half years after resection of the tumor, secondary sexual characteristics progressed with regression of tumor markers, and no mass was noted on ultrasonography, leading to the suspicion of central precocious puberty. Pubertal basal luteinizing hormone (LH) and elevated triptorelin-stimulated LH confirmed the diagnosis of central precocious puberty secondary to granulosa cell tumor of the ovary.

## Introduction

Secondary central sexual precocity is a rare cause of sexual precocity which has been described in association with late treatment initiation in CYP21 deficiency, leading to a simple virilizing form of congenital adrenal hyperplasia, testotoxicosis, McCune-Albright syndrome, and sex cord tumor of the ovary. Of these, ovarian sex cord tumor leading to secondary central precocious puberty is extremely rare [1]. Here, we report a case of isosexual peripheral sexual precocity due to granulosa cell tumor of the ovary transforming to central sexual precocity secondary to treatment in a five-year and five-month-old girl.

## Case presentation

**Figure 1:**
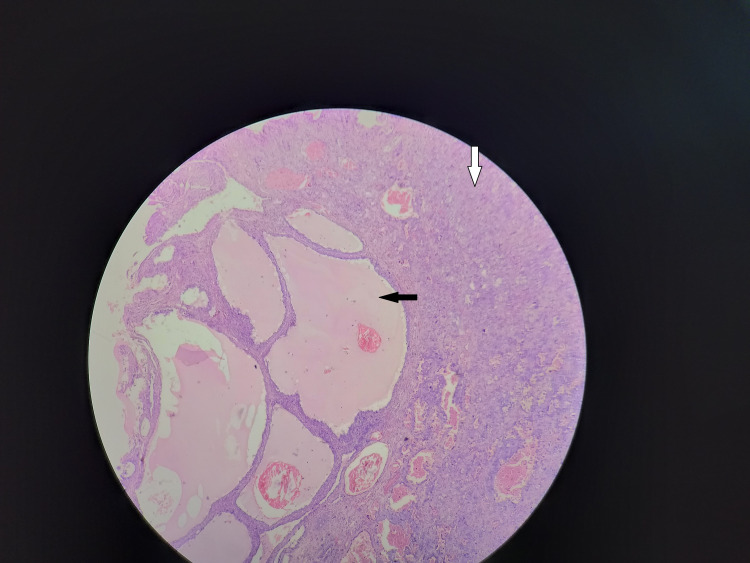
Histopathology: the black arrow indicates cells arranged in tubulocystic pattern, and the white arrow indicates solid sheet pattern.

**Figure 2:**
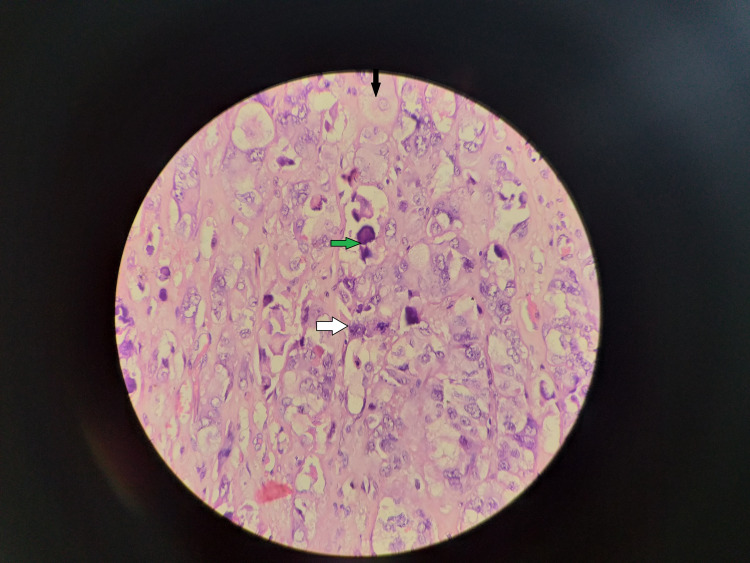
Histopathology: the black arrow indicates eosinophilic cytoplasm, the green arrow indicates basophilic mucin secretions, and the white arrow indicates mitotic figure.

A five-year and five-month-old girl presented with a history of progressive enlargement of bilateral breasts and intermittent vaginal spotting every four to seven days for the last two and a half years. It was initially associated with growth acceleration but not with axillary and pubic hair development. The patient did not have any history of headache, vomiting, visual disturbance, or seizure. There was no abnormal bony growth or café au lait patches over the skin. She was initially evaluated by a gynecologist, and her hormonal investigations suggested isosexual peripheral precocious puberty (Table 1). Ultrasonography of her pelvis showed a uterine volume of 6 ccs, uterine size 3.6 × 1.2 × 2.6 cm with right adnexal space-occupying lesion measuring 9.46 × 9.2 × 6.4 cm, which was suggestive of an ovarian tumor. Subsequently, she underwent laparotomy with tumor resection. Histology revealed large atypical cells arranged in tubulocystic and solid sheet pattern with nuclear atypia and eosinophilic cytoplasm in association with basophilic mucin secretion and mitotic figure, which was suggestive of juvenile granulosa cell tumor (Figures 1, 2).

**Table 1:** Preoperative and postoperative hormonal parameters and post-triptorelin stimulation test findings. LH: luteinizing hormone; FSH: follicle-stimulating hormone; CA 125: cancer antigen 125; LDH: lactate dehydrogenase

	Hormonal parameters				
	LH (IU/L)	FSH (IU/mL)	Estradiol (pg/mL)	CA 125 U/mL (0–35)	LDH U/L (140–280)
Preoperative	0.28	0.81	21.10	48.31	310
Postoperative	0.70	3.6	11	15.2	207
Triptorelin stimulation	Basal LH	60-minute LH	180-minute LH		
Preoperative	0.01	2.2	1.7		
Postoperative	0.01	5.2	5.2		

Immunohistochemistry was positive for inhibin, vimentin, calretinin, CD56, and CD99, confirming the diagnosis of juvenile granulosa cell tumor. Thereafter, she received chemotherapy with cisplatin, etoposide, and bleomycin.

After one and a half years of surgery, the tumor markers had declined but breast development progressed. Moreover, intermittent vaginal spotting persisted but frequency decreased to every 15 to 20 days. At this point in time, the patient was referred to Endocrinology Department.

**Figure 3:**
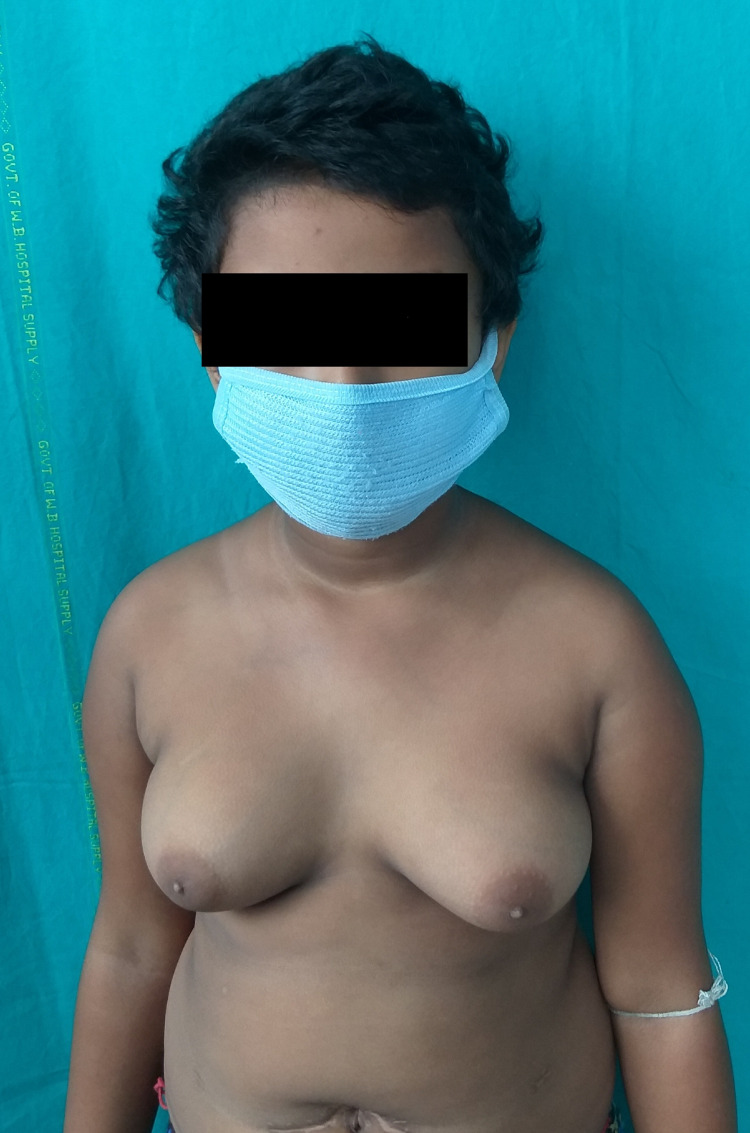
Clinical picture showing bilateral breast enlargement: Tanner stage 3.

**Figure 4:**
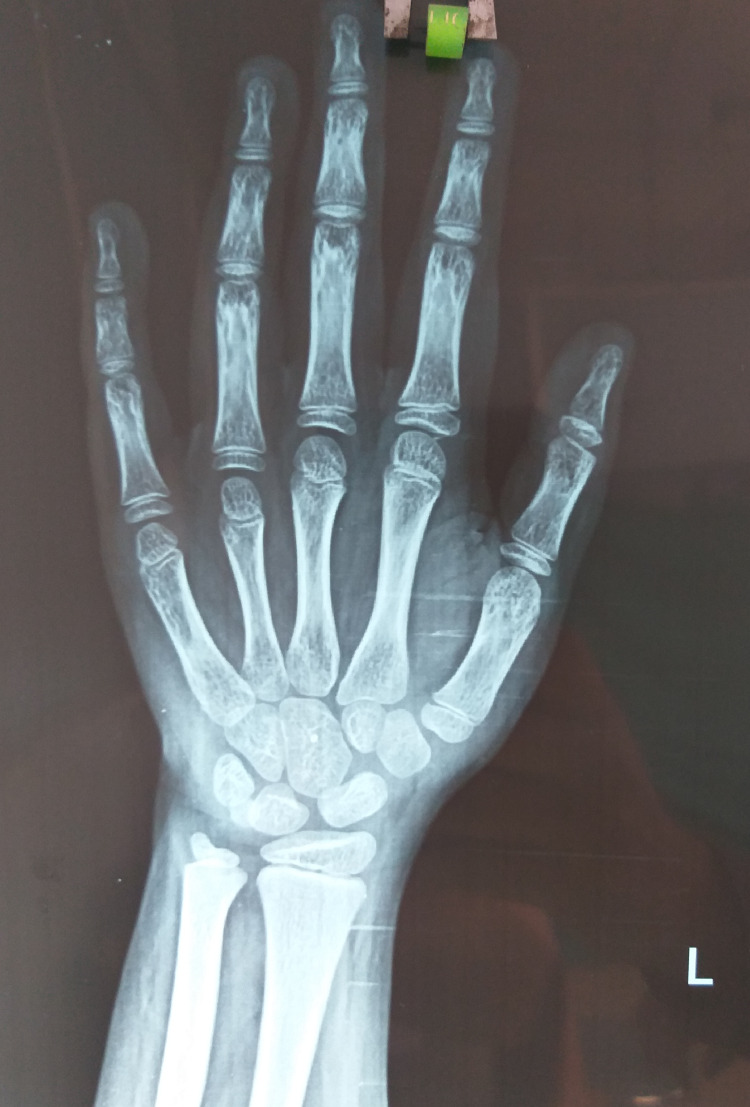
X-ray of the left hand showing advanced bone age.

On presentation to our department, the patient had a height of 121 cm corresponding to 90-97th percentile for her age and +1.81 SD, her weight was 24 kg corresponding to +1.71 SD, her breast was Tanner stage 3 (Figure 3), while her bone age had advanced to 11 years (Figure 4). Her hormonal evaluation at this time suggested central precocious puberty (Table 1). Postoperative ultrasonography of the pelvis showed no mass lesion and the tumor markers (cancer antigen 125, lactate dehydrogenase) were within normal limits, ruling out tumor recurrence. Treatment was initiated with injection leuprolide depot 11.25 mg every three months which led to stoppage of vaginal spotting and gradual regression of breasts.

## Discussion

Precocious puberty is defined as the development of secondary sexual characteristics before the age of eight in girls and nine in boys [2]. The overall incidence of sexual precocity is estimated to be 1:5,000 to 1:10,000 children with a female preponderance (female-to-male ratio may be as high as 10:1) [3]. Central precocious puberty secondary to chronic exposure to sex steroid hormones is a rare cause of central precocious puberty. It can occur in cases of late treatment of simple virilizing congenital adrenal hyperplasia, tumors secreting sex steroid hormones, testotoxicosis, McCune-Albright syndrome, or endocrine disruptors [1]. Long-term exposure to sex steroids leads to early maturation of the hypothalamic center required for the initiation of puberty, whereas treatment of the primary disease leads to a decrease in sex steroid hormones and subsequent activation of the primed gonadotropin-releasing hormone (GnRH) pulse generator [4]. In our patient, secondary sexual characters were progressing despite resection of granulosa cell tumor and postoperative chemotherapy, with declining postoperative tumor markers leading to suspicion of central precocity secondary to granulosa cell tumor.

During childhood, ovarian tumors are uncommon and rarely lead to precocious puberty. Sex cord tumor constitutes 8% of all ovarian tumors, and granulosa cell tumors fall under this category [5,6]. Precocity is the most common presentation in prepubertal girls [6], whereas abdominal mass, abdominal pain, and virilization are the presenting features in postpubertal girls [7]. Serum estradiol concentration is elevated in most of these patients [8]; however, 30% of granulosa cell tumors do not produce estradiol because of the lack of theca cells in tumor stroma. Because fluctuating levels of estradiol have been found in patients, estradiol levels are not a reliable marker of disease activity. Estradiol may serve as a reliable tumor marker for granulosa cell tumors of the ovary, but it is not sensitive enough to serve as a tumor marker [9]. On histopathological examination, granulosa cell tumor reveals a macrofollicular growth pattern with eosinophilic cytoplasm, round nuclei, and mitotic figures. They stain positively with inhibin, vimentin, CD99, and reticulin. Juvenile granulosa cell tumors have a highly favorable prognosis after surgical resection [10]. In our patient’s histological findings, immunohistochemistry and tumor markers were indicative of juvenile granulosa cell tumor of the ovary.

Central precocious puberty is diagnosed by basal pooled LH value of >0.3 IU/L using modern ultrasensitive automated chemiluminescence assays. GnRH stimulation test is performed using triptorelin 100 µg/m^2^ or leuprolide 20 µg/kg [11,12], and a post-stimulation LH of >5 IU/L after 30-60 minutes of stimulation is considered a positive result. Peak LH/peak FSH of >1 (>0.6 in some studies) also indicates true central precocious puberty. Uterine length of >3.5 cm and uterine volume of >1.8 mL are specific indicators of precocious puberty. Indications for therapy in children with a diagnosis of central precocious puberty are serum estradiol of >10 pg/mL, the rapid advancement of height and bone age, parental anxiety, and psychological stress. Treatment options are 3.75 mg monthly, 11.25 mg three-monthly injectable leuprolide or triptorelin, or 10.8 mg three-monthly goserelin [12,13].

## Conclusions

Juvenile granulosa cell tumor leading to isosexual peripheral precocious puberty is a rare but known entity. We have reported an even rarer case of central precocious puberty secondary to the resection of granulosa cell tumor, which had initially presented with features of isosexual peripheral precocious puberty. This case highlights the necessity for close follow-up of similar patients.
